# Persistent Parosmia Caused By COVID-19 Infection: An Emerging Symptom

**DOI:** 10.7759/cureus.19921

**Published:** 2021-11-26

**Authors:** Wael Khalifah, Basem Damanhouri, Bandar Abushal, Osama Marglani, Bashaer Alharbi, Murouj Almaghrabi, Rawan Alosaimy, Ahaad M Basahal

**Affiliations:** 1 Otolaryngology, Jeddah University, Jeddah, SAU; 2 Otolaryngology, King Abdullah Medical City, Makkah, SAU; 3 Otolaryngology, Ministry of Health, Makkah, SAU; 4 Otolaryngology-Head & Neck Surgery, King Faisal Specialist Hospital & Research Center, Jeddah, SAU; 5 Medicine, College of Medicine, Umm Al-Qura University, Al-Abdia Main Campus, Makkah, SAU

**Keywords:** olfactory dysfunction, covid-19, taste dysfunction, anosmia, parosmia

## Abstract

Objectives

Coronavirus disease 2019 (COVID-19) is associated with several known signs and symptoms, including olfactory disturbances leading to anosmia and parosmia. This study aimed to evaluate the clinical features of the emerging symptoms of post-COVID-19 parosmia and to report the outcome of commonly used intervention methods.

Methods

The study’s sample included post-COVID-19 patients who complained of parosmia, presented to otorhinolaryngology clinics at private tertiary care hospitals from December 2020 to April 2021. Patients’ data were collected from the hospital system and through direct phone interviews. The Modified Arabic 20-Items Sino-Nasal Outcome Test (MA-SNOT-20) was used to measure the severity of rhinosinusitis symptoms.

Results

Twenty-one patients were included in the study, and their ages mainly ranged between 20 and 39 years (76.2%), predominantly females 19 (90.5%). Post the COVID-19 illness course, nearly half of the patients (9; 42.9%) reported full recovery of olfaction and taste senses. Nine cases (42.9%) received treatment with intranasal and oral steroids, of which only three cases (14.3%) noticed improvement. The remaining 12 cases (57.1%) did not receive any treatment, two of which improved (9.5%). The maximum MA-SNOT score was 37 while the minimum was 3, and eight patients (38.1%) scores were between one and 10 points.

Conclusion

Olfactory and taste dysfunctions are common symptoms of COVID-19. The emerging symptom of parosmia is worth reporting in the literature to increase the awareness of this particular symptom in this pandemic era. Many management strategies have been introduced that might be effective. However, further studies are needed to establish evidence-based management protocols.

## Introduction

Severe acute respiratory syndrome coronavirus 2 (SARS-CoV-2) is a novel coronavirus variant that is highly contagious and responsible for the current ongoing pandemic called coronavirus disease (COVID-19) [1]. Several nonspecific signs and symptoms are observed in patients with COVID-19 infection. However, the most common clinical manifestations are fever, dry cough, shortness of breath, headache, and myalgia. Other atypical manifestations include anosmia (loss of smell) and ageusia (loss of taste) as isolated symptoms [2-4].

Olfactory disturbances are divided into quantitative and qualitative impairments: quantitative as hyposmia and anosmia (decline of sensitivity) and qualitative as parosmia and phantosmia of smelling hallucination (the odor is mostly unpleasant). Parosmia and phantosmia (perversion of sense of smell) that presents the sense of a bad smell not related to a specific odor exposure, as affected individuals would smell odors absent from their surrounding environment [5-7].

The Modified Arabic 20-Items Sino-Nasal Outcome Test (MA-SNOT-20) is a validated Arabic translated version of the Sino-Nasal Outcome Test (SNOT) [8]. This instrument is mainly intended to assess the efficacy of treatment, as it measures the severity of rhinosinusitis symptoms across different domains, including physical issues, functional limitations, and emotional consequences. It has been used in several previous studies and could be beneficial in patients with COVID-19 [8-12].

Multiple recent studies investigated olfactory and taste dysfunctions as they are a common symptom of COVID-19 infection and the correlation between COVID-19 and anosmia, as well as parosmia, as the most presenting symptoms, if not the only symptoms [13-15]. Parosmia is considered an emerging symptom especially if associated with COVID-19. Additionally, the Ear, Nose, and Throat (ENT) Society of the United Kingdom (UK) and British Rhinological Society reported an anecdotal relationship between olfactory dysfunction and COVID-19 infection, especially parosmia [16]. A recent systematic review and meta-analysis on 3563 patients confirmed the significance of this relationship and recommended self-isolation for patients complaining of smell or taste impairment [17]. Besides, parosmia and phantosmia were recorded in 57 and 38 of 78 COVID-19 patients during the treatment course, respectively [18], while another investigation observed these symptoms in 34% and 20% of patients, respectively [19].

However, studies are still lacking regarding these symptoms and their management, especially in Saudi Arabia. To our knowledge, only one published case reported persistent parosmia not responding to treatment after COVID-19 infection. Thus, our study aimed to evaluate the clinical outcomes of post-COVID-19 parosmia and to report the outcome of commonly used intervention methods among 21 cacosmia cases presented to our clinic.

## Materials and methods

Inclusion and exclusion criteria 

The study’s population sample was chosen from patients who attended ENT clinics at a private tertiary care hospital from December 2020 to April 2021. Our study included patients of any age who had a history of COVID-19 infection with positive polymerase chain reaction (PCR) results and complained of parosmia or anosmia followed by parosmia. Any patient with a previous history of smell or taste problems (before COVID-19) was excluded from the study.

Assessment tool and interviews

According to the literature, no validated instrument has been introduced for assessing the parosmia symptom, especially among COVID-19 patients. Therefore, the patient’s description of the parosmia symptom was the only way depended on for reporting the current study.

The MA-SNOT score was used as a symptomatic measurement tool [8], which assessed 16 sino-nasal symptoms, ranging from 0 to 5 for each symptom, according to severity, with a total score of 80. Patients’ data were collected from the hospital information system (HIS) and individual contact. The MA-SNOT score was recorded for each patient at the time of presentation to the clinic. Additionally, each patient was asked about any undertaken intervention modality, including intranasal steroid sprays, oral steroids, or olfactory rehabilitation methods, and the changes after the intervention, including recovery rate. The interviews were conducted by phone calls and were completed in April 2021.

Ethical approval and consent to participate

This study was reviewed and approved by the institutional review board (IRB) of the International Medical Center (IMC), Jeddah, SA (approval number: 157). Verbal consent was obtained from each patient prior to answering the questions, and confidentiality was assured. Names and personal information were not requested from any patient to ensure anonymity.

Reviewed variables and statical analysis

Demographic characteristics, including age, gender, marital status, smoking history, and past medical history, were recorded. Questions were asked about parosmia characteristics, including data about the impairment sensation, duration of parosmia, status of anosmia, and treatment outcomes if any treatment was received. Additionally, MA-SNOT scores were also noted. Data were analyzed using IBM SPSS-26 software (IBM Corp. Armonk, NY). The continuous data were expressed as mean ± standard deviation while frequencies and percentages were used for all categorical variables.

## Results

Characteristics and clinical course of the study population

The study included 21 individuals with positive COVID-19 diagnoses (mean age 34.4 ± 9.7 years) who presented to the clinic with parosmia complaints. The study participants were mostly females 19 (90.5%), all of them were non-pregnant. Concerning other known risk factors for severity of COVID-19 illness, the selected population was diverse in terms of age, smoking status, and past medical history, as most of them 16 (76.2%) were in the young adult age group (20-39 years), non-smokers 17 (81.0%), and healthy 12 (57.1%). All included patients had positive PCR-based diagnoses from nasal swabs for SARS-CoV-2. During the course of the COVID‐19, all 21 patients (100%) reported having chemosensory dysfunctions in the form of loss of olfaction and taste, followed by body ache (18; 85.7%) (Table 1).

**Table 1:** Socio-demographics and clinical features of the patients (n=21) *The recorded respiratory conditions were asthma, allergic rhinitis, and sinuses. **Endocrine conditions: DM and hypothyroidism. *** Other conditions were hypertension (HTN), urticaria, paroxysmal supraventricular tachycardia, and migraine headache.

Socio-demographic data		No	%
Gender	Male	2	9.5%
	Female	19	90.5%
Age (years)	19 years	1	4.8%
	20-39	16	76.2%
	40+	4	19.0%
Marital status	Single	5	23.8%
	Married	16	76.2%
Smoking status	Smoker or passive smoker	4	19.0%
	Non-smoker	17	81.0%
Past medical history	Respiratory condition *	4	19.0%
	Endocrine condition **	4	19.0%
	Others ***	5	23.8%
	None	12	57.1%
COVID-19 symptoms	Loss of taste and smell	21	100%
	Body ache	18	85.7%
	Fever	16	76.2%
	Rhinorrhea or nasal congestion	10	47.6%
	Headache or dizziness	8	38.1%
	Sore throat	6	28.6%
	Cough	6	28.6%
	Gastrointestinal symptoms	5	23.8%

**Figure 1:**
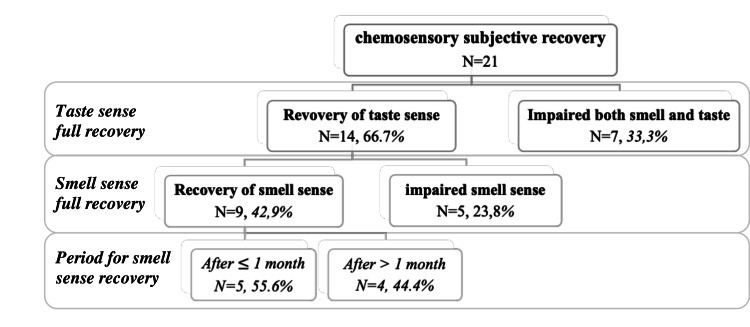
Chemosensory subjective recovery post-COVID-19 patients (n=21)

After COVID-19 illness, nearly half of the patients 9 (42.9%) reported full recovery of both olfaction and taste. Looking into the sense’s recovery separately, more than half of the cases (14; 66.7%) reported full taste recovery, among them 9 (42.9%) had full smell recovery. The smell sense recovery occurred in one month or less in five cases (55.6%) while the remaining four cases (44.4%) needed more than one month to recover. Later, all included cases presented to the clinic complaining of alteration in the olfaction in form of parosmia (Figure 1).

The period between onset of parosmia symptom differed widely amongst patients, as some of them had complete recovery of the smell sensation. In contrast, others didn’t improve, and their anosmia turned into parosmia. For those cases who had a full recovery of smell, the duration of normal smell ranged from one month in four cases (19%), to two to four months in four cases (19%), and up to seven months in one case (4.8%).

Characteristics of the patients’ complaints (parosmia)

Parosmia was reported by all patients, with a duration ranging from one month to eight months in some cases (Table 2).

**Table 2:** Duration of the parosmia in patients with a previous history of COVID-19 (n=19)

Variables		No	%
Duration of parosmia	3 months	11	52.4%
	> 3 months	10	47.6%
	Minimum = 1 month		
	Maximum = 8 months		

Parosmia was bilateral, continuous, without discharge, and the smell was not noticeable in their surroundings among all the cases. Nearly all patients described it as an unpleasant foul odor. Some of them could not describe it while others described it as resembling the smell of musty food, moldy fish, matches, or plastic balloons. It was aggravated mainly by potent odors, such as citrus aroma, perfumes, and cleaners, along with the smell of some types of food such as garlic, onion, cardamom, chicken, and meat. However, few cases reported no aggravating factors.

**Figure 2:**
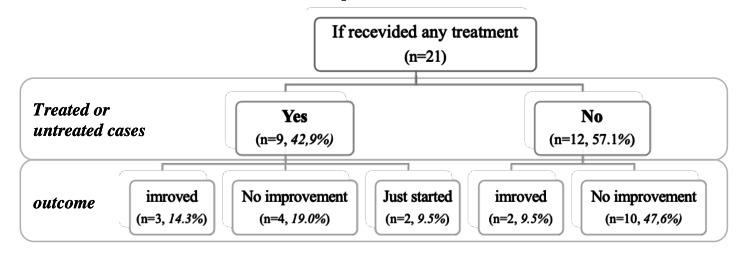
The number of treated parosmia cases and their clinical outcomes among post-COVID-19 patients (n=21)

All patients had normal clinical examination (with no nasal obstruction), imaging, and laboratory findings. The time interval between the onset of parosmia and the start of treatment varied amongst individuals, depending on the duration of parosmia at the time of presentation to the clinic. As shown in Figure 2, nine cases (42.9%) received treatment with intranasal and oral steroids; only three cases (14.3%) noticed improvement. The remaining 12 cases (57.1%) didn’t receive any treatment, and only two (9.5%) improved.

MA-SNOT scores of the patients

MA-SNOT scores were obtained from each patient, with a mean of 18.14 ± 10.99. The maximum recorded score was 37 while the minimum score was 3. Eight patients had MA-SNOT scores between 1 and 10 points (38.1%). At the same time, five patients had scores between 21 and 30 points (23.8%). Other score categories were reported equally by four patients in each (for 11-20 points and 31-40 points) (Table 3).

**Table 3:** MA-SNOT results of post-COVID-19 parosmia patients (n=21) MA-SNOT: Modified Arabic 20-Items Sino-Nasal Outcome Test

Variables		No	%
MA-SNOT scores	1-10 points	8	38.1%
	11-20 points	4	19.0%
	21-30 points	5	23.8%
	31-40 points	4	19.0%
Loss of sense of taste/smell (Item in MA-SNOT)	No problem	3	14.3%
	Mild or slight problem	3	14.3%
	Moderate problem	3	14.3%
	Severe problem	4	19.0%
	Problem as bad as it can be	8	38.1%

Within MA-SNOT, the patients were asked to rate the taste/smell loss severity in scores ranging from 0 to 5. Eight patients reported that ‘problem as bad as it can be (38.1%), followed by ‘severe problem’ reported by four patients (19%). An equal result was recorded in the other three categories: mild, moderate, and no problems (3 patients; 14.3% for each category).

## Discussion

Among our included cases, we noticed that taste and smell dysfunctions were more frequent among female patients, adulthood age, non-smokers, non-pregnant, and without past medical history. Concerning their COVID-19 infection, they lost taste and smell sensation, with fever and body aches in most of them along with other nonspecific symptoms. All of them were isolated at home. The taste and smell dysfunctions were severely affected rather than mild-moderate. Most of the patients experienced a restored taste sensation after COVID-19 infection recovery. In contrast to anosmia, in which half of the patients recovered completely before developing parosmia. This study demonstrates that parosmia duration ranges from less than two months to up to more than seven months. Most of them shared the same aggravating factors as potent odors such as perfumes, soap, and cleaners. Examination and investigations were normal in all of them. Only three out of nine patients who received treatment noticed some improvements after treatment, but the smell wasn't fully restored until writing this report.

A previously published modified Arabic version of the MA-SNOT score has been used to assess the severity of the symptoms among outpatients [8]. Based on MA-SNOT, the score of healthy individuals is 2.6±3.7, and the score of diseased individuals is 30.9±14.3; the higher the number, the higher the severity [8]. In the current study, 38.1% of patients reported a score of 1-10, 23.8% reported a score of 21-30, and 19% reported 31-40, which was considered severe.

One of the most common causes of olfactory dysfunction in adults is post-viral olfactory dysfunction [20], with coronaviruses accounting for 10%-15% of cases [21]. A previous study hypothesized that olfactory dysfunction in COVID-19 patients is secondary to mucosal obstruction of the olfactory cleft or directly affecting olfactory mucosa and the sensory neurons, leading to a sensorineural loss [20]. It is corroborated by our observation that none of the patients had nasal blockage during the examination. However, some patients showed complete restoration of smell or taste sensation, similar to a recent prospective study [20]. Furthermore, the persistence of the impairment was not associated with a persistent COVID-19 infection, as all patients reported negative PCR test results.

At present, to our knowledge, only one case report has been published regarding cacosmia by Okar et al. The patient was a female, pregnant (G3, P2) with no previous history of any allergic respiratory diseases. She was diagnosed with COVID-19, and after recovery, the only remaining symptom was persistent smell disturbance. Her cacosmia was bilateral, continuous, without discharge, and aggravated by potent odors (perfumes, soaps, or cleaners). ENT examination revealed pale nasal mucosa, and her lab investigations results were normal. She received an intranasal corticosteroid (Mometasone), but unfortunately, it did not improve the symptoms and she was still complaining of cacosmia [6].

There are noticeable similarities between the current study’s cases and previously published case reports. First, the gender and age distribution of the cases showed that 95.2% of the cases were female, of adulthood age. According to the literature, most studies suggested that the incidence of smell disorders among COVID-19 patients is higher in females than males [22-24]. Other reportedly associated factors were younger age [24] and having fever and gustatory dysfunctions [22]. Other similarities included the absence of smell disturbance improvement after COVID-19 recovery; the cacosmia character and aggravating factors were almost similar between the cases. In addition, all their investigation findings were within the normal range (except for a patient who had low vitamin B12 levels), and the smell sensation was not restored in all treated cases. However, some differences exist between the current study’s cases and the previously reported cases in the literature. The most important difference was in the ENT examination, as the patient had pale nasal mucosa, whereas our cases were all within normal range. Also, in that study, the patient used an intranasal corticosteroid but experienced no improvement. While an improvement was reported in three cases in the current study, but four cases showed no improvement, two just started treatment, two cases showed improvement without treatment, and 10 showed no improvement and received no treatment.

Hopkins et al. investigated the most preferred treatment for patients with COVID-19 who had a new onset of smell loss and recommended against the use of alpha-lipoic acid. On the other hand, they recommended olfactory training for all patients with more than two weeks of smell loss. Moreover, oral steroids, steroid rinses, and omega-3 supplements were considered individually [25].

The prognosis of olfactory dysfunction following COVID-19 infection is currently being studied, and more research is needed to identify its prognosis and the best effective therapeutic techniques. Generally, in post-infectious olfactory dysfunction, spontaneous recovery is observed within one to three years among 32%-66% of patients [26]. The mechanism can be explained by spontaneous regeneration, as various investigations have demonstrated [26-28]. Regarding short-term olfactory recovery among COVID-19 patients, previous data in Europe showed a recovery rate of 44%; among them 72.6% recovered within the first week [22]. Nevertheless, a significant percentage of patients still suffered an olfactory disorder affecting their lives, such as cacosmia, presented in this study.

## Conclusions

The current study presented 21 patients with taste and smell dysfunctions, where most of the included patients were female, of adulthood age, non-smokers, non-pregnant, and without a past medical history of any known condition/illness. Most of them shared the same aggravating factors such as potent odors like perfumes, soap, and cleaners.

Olfactory and taste dysfunctions are common symptoms of COVID-19 infection. The emerging symptom of parosmia is worth reporting in the literature to increase the awareness of this particular symptom in this pandemic period. Many management strategies might be effective. However, more prospective randomized controlled trials are required to establish the cause-and-effect relationship and evidence-based management protocols.
